# Changes in Otorhinolaryngologic Disease Incidences before and during the COVID-19 Pandemic in Korea

**DOI:** 10.3390/ijerph192013083

**Published:** 2022-10-12

**Authors:** So Young Kim, Dae Myoung Yoo, Ji Hee Kim, Mi Jung Kwon, Joo-Hee Kim, Juyong Chung, Hyo Geun Choi

**Affiliations:** 1 Department of Otorhinolaryngology-Head & Neck Surgery, CHA Bundang Medical Center, CHA University, Seongnam 13496, Korea; 2 Hallym Data Science Laboratory, Hallym University College of Medicine, Anyang 14068, Korea; 3 Department of Neurosurgery, Hallym University College of Medicine, Anyang 14068, Korea; 4 Department of Pathology, Hallym University College of Medicine, Anyang 14068, Korea; 5 Division of Pulmonary, Allergy, and Critical Care Medicine, Department of Medicine, Hallym University College of Medicine, Anyang 14068, Korea; 6 Department of Otorhinolaryngology-Head & Neck Surgery, Wonkwang University School of Medicine, Iksan 54538, Korea; 7 Department of Otorhinolaryngology-Head & Neck Surgery, Hallym University College of Medicine, Anyang 14068, Korea

**Keywords:** COVID-19, communicable diseases, influenza, human, respiratory tract infections, risk factors

## Abstract

This study aimed to investigate the change in the incidence and variance of otorhinolaryngologic diseases during the coronavirus disease 19 (COVID-19) pandemic. The entire Korean population (~50 million) was evaluated for the monthly incidence of 11 common otorhinolaryngologic diseases of upper respiratory infection (URI), influenza, acute tonsillitis, peritonsillar abscess, retropharyngeal and parapharyngeal abscess, acute laryngitis and bronchitis, stomatitis and related lesions, acute sinusitis, rhinitis, otitis media, and dizziness from January 2018 through March 2021 using the International Classification of Disease (ICD)-10 codes with the data of the Korea National Health Insurance Service. The differences in the mean incidence of 11 common otorhinolaryngologic diseases before and during COVID-19 were compared using the Mann—Whitney U test. The differences in the variance of incidence before and during COVID-19 were compared using Levene’s test. The incidence of all 11 otorhinolaryngologic diseases was lower during COVID-19 than before COVID-19 (all *p* < 0.05). The variations in disease incidence by season were lower during COVID-19 than before COVID-19 for infectious diseases, including URI, influenza, acute tonsillitis, peritonsillar abscess, retropharyngeal and parapharyngeal abscess, acute laryngitis and bronchitis, acute sinusitis, and otitis media (all *p* < 0.05), while it was not in noninfectious diseases, including stomatitis, rhinitis, and dizziness. As expected, the incidences of all otorhinolalryngolgic diseases were decreased. Additionally, we found that seasonal variations in infectious diseases disappeared during the COVID-19 pandemic, while noninfectious diseases did not.

## 1. Introduction

Coronavirus disease 19 (COVID-19) is an infectious disease caused by severe acute respiratory syndrome coronavirus 2. COVID-19 has changed our daily lives. Due to the limitations of accessibility of clinics, the clinical procedures were preferentially provided to those with emergency or acute phases of the disease [1,2]. Medical resources have been redistributed to triage emergent diseases. For instance, in neurosurgery departments, the proportion of elective surgery decreased from 57.7% in the pre-COVID-19 era to 11.3% in the COVID-19 era (*p* < 0.001) [2]. Both research and clinical practice have been focused on COVID-19. Thus, there has been concern about the inequality of medical care [3]. In addition to changes in medical resources, the epidemiologic features of diseases have changed during the COVID-19 pandemic period. Changes in health-seeking behavior and improved hygiene have an impact on the incidence of disease [4]. A healthy diet and lifestyle habits were suggested to decrease the incidence of metabolic diseases as well as infectious diseases [5]. In Korea, nationwide surveillance strategies have been acted for the early detection and diagnosis of patients with COVID-19 [6]. In addition, social distancing strategies have been continued to prevent the spread of COVID-19 [7].

Multiple factors determine the epidemic of infectious diseases, including circulating viruses, the immunity of the population, and the transmissibility of viruses [8,9]. Among these determinants, the transmissibility of viruses can be diminished during the COVID-19 pandemic period by strategies of quarantine. Due to changes in health-seeking behavior and improved hygiene, the distribution of disease incidence as well as the overall incidence of diseases can change during the COVID-19 pandemic period. A number of recent studies have suggested alleviating the burden of respiratory infectious diseases during COVID-19. Influenza contraction declined by approximately 44% (95% confidence intervals = 34%–53%) during social distancing measures against the COVID-19 pandemic in Hong Kong [4]. As otorhinolaryngologic diseases include many upper respiratory infectious diseases, their incidence and epidemics could be influenced by the COVID-19 pandemic. A retrospective study reported a decrease in overall otorhinolaryngological diseases (80.4% vs. 19.5%, *p* < 0.001) and otitis media (92.8% vs. 8.0%, *p* < 0.001) during the lockdown period (21 February–3 May 2020) in Italy [10]. However, the study population was limited to children, and the study population was small (4538) [10]. In addition, there has been no previous research on the variance or seasonality of infectious diseases during COVID-19.

We hypothesized that the incidence and variance of a wide range of otorhinolaryngologic diseases, in addition to influenza and upper respiratory infection (URI), may be changed during the COVID-19 era. To test this hypothesis, this study compared the number and variations of incidence of numerous otorhinolaryngologic diseases between the pre-COVID-19 pandemic and during the COVID-19 pandemic period. To estimate the seasonality of diseases, the incidences of diseases were collected monthly and compared between the pre-COVID-19 pandemic and the COVID-19 pandemic period. Few previous studies have extensively investigated the impact of the COVID-19 pandemic on the epidemiology of otorhinolaryngological diseases in a large, nationwide population.

## 2. Methods

### 2.1. Ethics

The Ethics Committee of Hallym University (code number: 2021-11-004) approved the use of these data. The study was exempted from the need for written informed consent by the Institutional Review Board.

### 2.2. Study Population

This study includes the entire Korean population (~50 million) without an exception, as a single, mandatory health insurance system covers the whole country. Thus, we could gather data on the entire Korean population from the primary clinic to the tertiary hospital. In this study, we evaluated the incidence of diseases from January 2018 through March 2021. As the first COVID-19 cases in Korea were discovered on 20 January 2020, and disease prevention and control were started on March 2020, we defined the periods of “before COVID-19” until February 2020 and “during COVID-19” from March 2020 to March 2021.

### 2.3. Otorhinolarynologic Diseases

We evaluated the monthly incidence of 11 otorhinolaryngologic diseases that are common in the primary clinic. The patients were diagnosed with each disease and recorded using the International Classification of Disease (ICD)-10 codes: URI (J00, J02, and J03), influenza (J09, J10, and J11), acute tonsillitis (J03), peritonsillar abscess (J36), retropharyngeal and parapharyngeal abscess (J39.0), acute laryngitis and bronchitis (J04), stomatitis and related diseases (K12), acute sinusitis (J01), rhinitis (J30), otitis media (H65, H66, and HJ67), and dizziness (R42 and H81). The incidence of these diseases was calculated without duplication, as we had the medical records of the entire hospital or clinics, and patients were identified with unique resident registration numbers.

### 2.4. Statistical Analysis

The difference in the mean incidence of diseases before and during COVID-19 was compared using the Mann—Whitney U test for nonparametric values. The difference in the variance of diseases before and during COVID-19 was compared using Levene’s test for nonparametric values [11]. For the subgroup analyses, we divided the participants by age (0–19 years old, 20–59 years old, and 60+ years old) and sex. The subgroup analyses were conducted due to the potential age and sex differences in terms of the impact of COVID-19. For instance, the knowledge of COVID-19 and quarantine maneuvers can have different impacts on daily life according to age and sex.

Two-tailed analyses were conducted, and *p* values < 0.05 were considered to indicate significance. The results were statistically analyzed using SPSS version 22.0 (IBM, Armonk, NY, USA).

## 3. Results

### 3.1. Incidences of Infectious Diseases

The incidence of URI, influenza, acute tonsillitis, peritonsillar abscess, retropharyngeal and parapharyngeal abscess, acute laryngitis and bronchitis, acute sinusitis, and otitis media was lower during COVID-19 than before COVID-19 (all *p* < 0.001, Table 1).

**Figure 1:**
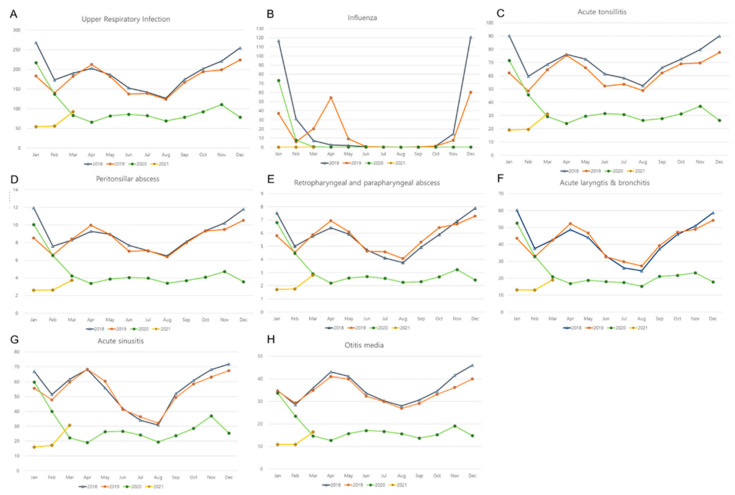
Monthly incidences of (**A**) upper respiratory infection, (**B**) influenza, (**C**) acute tonsillitis, (**D**) peritonsillar abscess, (**E**) retropharyngeal and parapharyngeal abscess, (**F**) acute laryngitis and bronchitis, (**G**) acute sinusitis, and (**H**) otitis media. (*y*-axis: ×10,000 person).

The variations in disease incidence were significantly lower during COVID-19 than before COVID-19 for all of these infectious diseases (all *p* < 0.05). All of these diseases demonstrated seasonality before COVID-19 (Figure 1). The incidences of URI and influenza were high during autumn and winter (October, November, December, January, and February) before COVID-19 (Figure 1). The incidences of acute tonsillitis, peritonsillar abscess, retropharyngeal and parapharyngeal abscess, acute laryngitis and bronchitis, acute sinusitis, rhinitis, and otitis media showed small peaks during spring (March, April, and May), in addition to the peak incidence during autumn and winter. During COVID-19, the seasonal variations in the incidence of all of these diseases were attenuated.

In terms of sex, both men and women showed a lower incidence of URI, influenza, acute tonsillitis, peritonsillar abscess, retropharyngeal and parapharyngeal abscess, acute laryngitis and bronchitis, acute sinusitis, and otitis media during COVID-19 than before COVID-19 (all *p* < 0.05, Table 2).

The variations in disease incidence were different before and during COVID-19 for URI, acute tonsillitis, peritonsillar abscess, retropharyngeal and parapharyngeal abscess, acute laryngitis and bronchitis, acute sinusitis, and otitis media in the male group (all *p* < 0.05). In the female group, the variations in the incidence of URI, influenza, acute tonsillitis, peritonsillar abscess, retropharyngeal and parapharyngeal abscess, acute laryngitis and bronchitis, acute sinusitis, and otitis media were low during COVID-19 compared with before COVID-19 (all *p* < 0.05).

In terms of age, all age groups showed a lower incidence of URI, influenza, acute tonsillitis, peritonsillar abscess, retropharyngeal and parapharyngeal abscess, acute laryngitis and bronchitis, acute sinusitis, and otitis media during COVID-19 than before COVID-19 (all *p* < 0.05, Table 3). The variances in disease incidence were lower for URIs and acute tonsillitis, peritonsillar abscess, retropharyngeal and parapharyngeal abscess, acute laryngitis and bronchitis, and otitis media during the COVID-19 pandemic in all age groups (all *p* < 0.05). The variance in acute sinusitis was lower during the COVID-19 pandemic in the age groups of 20-to-59 years and 60 years or above (all *p* < 0.05).

### 3.2. Incidences of Noninfectious Diseases

**Figure 2:**
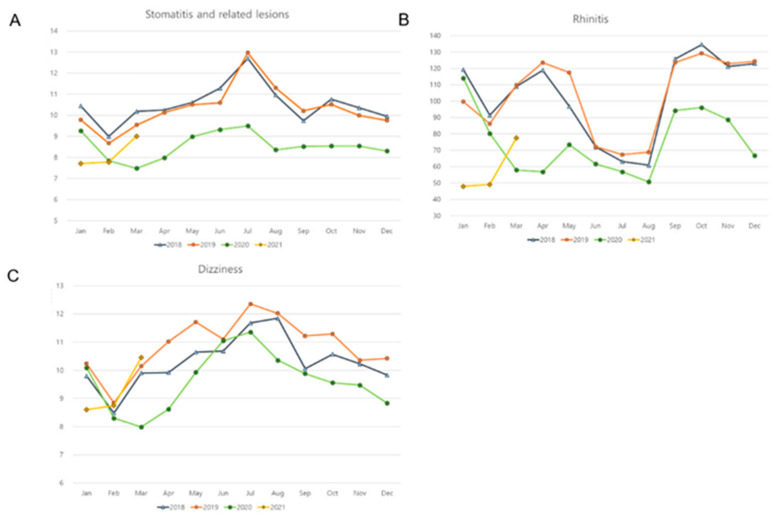
Monthly incidences of (**A**) stomatitis and related lesions, (**B**) rhinitis, and (**C**) dizziness. (*y*-axis: ×10,000 person).

Stomatitis and related lesions, rhinitis, and dizziness showed a lower incidence during COVID-19 than before COVID-19 (all *p* < 0.001, Table 1). These noninfectious diseases demonstrated no difference in variations in disease incidence before and during COVID-19. Dizziness showed a similar incidence of diseases throughout the year (Figure 2). The incidence of stomatitis and related lesions was high during the summer season (July and August) before COVID-19 (Figure 2). The incidences of rhinitis showed a small peak during spring (March, April, and May), in addition to the peak incidence during autumn and winter before COVID-19.

The subgroup analyses also demonstrated a lower incidence of stomatitis and related lesions, rhinitis, and dizziness during COVID-19 than before COVID-19 in both the male and female groups (all *p* < 0.05, Table 2). The variations in the incidence of stomatitis and related lesions, rhinitis, and dizziness were not significant before COVID-19 or during COVID-19 in the male and female subgroups, except for stomatitis and related lesions in the female subgroup. The female group showed a lower variance in the incidence of stomatitis and related lesions during COVID-19 than before COVID-19 (all *p* = 0.035).

In all the age subgroups, stomatitis and related lesions and rhinitis showed lower incidence during the COVID-19 pandemic (all *p* < 0.05, Table 3). The incidence of dizziness was lower during the COVID-19 pandemic in the 20-to-59-year-old group (*p* = 0.001). The variation in the incidence of stomatitis and related lesions, rhinitis, and dizziness was not different during the COVID-19 pandemic compared with before the COVID-19 pandemic in all age groups, except for the decreased variance in rhinitis in those 60 years old or older (*p* = 0.013).

## 4. Discussion

During the COVID-19 pandemic period, the incidences of otorhinolaryngologic diseases were lower than before the COVID-19 pandemic period. In addition, the seasonality of infectious diseases was attenuated during the COVID-19 pandemic period. On the other hand, noninfectious diseases, such as stomatitis, rhinitis, and dizziness, did not show differences in the variance in disease incidence during COVID-19. The decreased incidence of otorhinolaryngologic diseases and the variance in infectious diseases during COVID-19 were consistent in sex and age subgroups. This study is novel in demonstrating the decreased variance in the incidence of various otorhinolaryngologic infectious diseases during the COVID-19 pandemic period compared with before the COVID-19 pandemic period in the general population. An extensive number of upper respiratory infectious diseases of URI, influenza, acute tonsillitis, peritonsillar abscess, retropharyngeal and parapharyngeal abscess, acute laryngitis and bronchitis, acute sinusitis, and otitis media were evaluated. In addition, noninfectious diseases of stomatitis, rhinitis, and dizziness were investigated, which did not show a difference in the variance of incidence before COVID-19 and during COVID-19.

Similar to the present results, a number of prior studies described the low incidence of infectious diseases during the COVID-19 pandemic. Among these studies, many reported a decreased incidence of influenza during the COVID-19 pandemic [4,12,13,14,15]. A retrospective study demonstrated the short and low epidemic peak of influenza during the 2019–2020 season, compared with the 2018–2019 season, in Korea (5.9 vs. 6.3 influenza-like illnesses per 1000 outpatients and 19-week 1-day vs. 31-week 1-day duration) [16]. In addition to influenza, other infectious diseases have been suggested to have declined during the COVID-19 pandemic [12]. In China, notifiable infectious diseases were reduced by approximately 41.38% in 2020 compared with 2019 [12]. Among infectious diseases, influenza was the most decreased infectious disease in 2020, followed by hand–foot–mouth disease and other infectious diseases such as diarrhea [12]. Other lower respiratory tract infections, such as community-acquired pneumonia and hospital-acquired pneumonia, were also reported to be declined during the COVID-19 pandemic [17,18]. Respiratory coronavirus infections other than SARS-CoV-2 were suppressed during the lockdown period in Chinese children (0–24 months) [13]. During the COVID-19 lockdown period, otitis media was reported to have declined in children [19]. As many as 82.3% of the cases of children with otitis media were resolved during the lockdown period and monitored using telemedicine [19]. Thus, it can be presumed that the COVID-19 pandemic status may have a role in the low incidence of various infectious diseases in both upper and lower respiratory tracts.

Nonpharmaceutical interventions (NPIs) may have preventive effects for upper respiratory infectious diseases in addition to mitigating the spread of SARS-CoV-2 transmission [20]. NPIs can be defined as preventive strategies in addition to pharmacologic management to cope with infectious diseases [21]. For instance, the wearing of facemasks, hand hygiene, and social distancing strategies can be considered as NPIs. In personal aspects, self-protective practices include wearing facial masks, hand hygiene, cough etiquette, and voluntarily staying home during the COVID-19 pandemic period [20]. In social aspects, social distancing acts encompass school closures, work at home, telecommuting, and avoidance of mass public gatherings [22]. These NPIs could suppress the contraction of many upper respiratory viruses in addition to severe acute respiratory syndrome-coronavirus 2 (SARS-CoV-2) during asymptomatic periods, which can harbor considerably high viral loads [23]. In Hong Kong, the daily effective reproduction number for influenza decreased to 0.72 (95% CI = 0.70–0.74) after school closure from 1.28 (95% CI = 1.26–1.30) during school closure [4]. A systemic review estimated an approximately 23% decline in cumulative influenza attacks associated with workplace social distancing measures in the general population [22].

In addition to the decrease in incidence, infectious diseases showed a lower variance in disease incidence during the COVID-19 pandemic in the present study. In line with the present study, a retrospective observational study described the extraordinarily low incidence of respiratory syncytial virus infection during the winter season, while a high incidence of respiratory syncytial virus infection during the summer season in England during the COVID-19 pandemic [24]. They suggested that the lack of immunity to the respiratory syncytial virus during winter may induce an increase in the incidence of respiratory syncytial virus infection during the summer season [24]. As a result, the seasonality of respiratory infection was diluted. In addition to respiratory infections, acute otitis media in children has been reported to have seasonality due to variations in the viral transmissibility of respiratory syncytial virus, human metapneumovirus, and influenza, and its seasonal variation was diminished in the current study [25]. The high prevalence of upper respiratory tract infectious diseases during the winter season is attributed to a number of viral and host factors, including increased crowding and indoor activities and the cooling of the nasal epithelium [8,9]. Increased viral stability and transmissibility and weakened host immune responses could accelerate the high incidence of infectious diseases during winter [26]. In host factors, the low temperature of the nasal epithelium could decrease the immunities and defense mechanisms to fight against viral infections, such as mucociliary clearance and the phagocytic activity of leukocytes [8]. In addition, due to cold weather, indoor activities and opportunities for physical contact with viral carriers can be increased during the winter season [8,9]. Although some viral and host factors, such as viral stability and host immune systems, cannot be overcome by NPIs, strict NPIs to cope with SARS-CoV-2 contraction can dissolve the seasonal epidemics of other infectious diseases. 

As there are variations in the severity of the COVID-19 pandemic among countries, the current data should be interpreted with caution [27,28]. This study analyzed the entire Korean population (~50 million). The meteorological characteristics of Korea show four distinctive spring, summer, autumn, and winter seasons because Korea resides in the Northern Hemisphere. Thus, it is susceptible to seasonal infectious diseases. In Korea, the first patient with COVID-19 was reported on 20 January 2020 [29], and therefore, we defined before February 2020 as before the COVID-19 period. From February 2020, COVID-19 was persistently outbroken in Korea [29]. To cope with the COVID-19 pandemic, the Korean government enacted stratified social distancing policies [29]. Thus, the effects of quarantine could be high in Korea.

This study was based on the health claim data that classified the diseases according to diagnostic codes. Therefore, undiagnosed or subclinical diseases could be missed in the present study. However, in Korea, a national influenza surveillance system exists, and consequently, there may be few undiagnosed cases [30]. In addition, the accuracy of diagnosis was limited due to the inaccessibility of the results of laboratory tests. There may be heterogeneity in the severity and subtypes of diseases. For instance, the transmissibility and severity of influenza vary depending on the subtypes of influenza virus (H1N1, H2N2, H3N3, H5N1, and H7N9 for influenza A) [31]. To attenuate the bias from any possible misclassification of diseases, we analyzed two years (2018 and 2019) before the COVID-19 pandemic period. The distributions of disease incidence were similar between 2018 and 2019 (Figure 1). As this study analyzed 11 diseases, the bias from multiple comparisons could not be excluded. This study counted the cumulative cases of otorhinolaryngologic diseases without the trends of the daily cumulative numbers of patients treated for otorhinolaryngologic diseases before and during COVID-19. Segmented regression analysis can improve the current limitation. Further follow-up studies might be warranted to delineate the effect of the COVID-19 pandemic on the incidence and seasonality of upper respiratory infectious diseases.

## 5. Conclusions

The incidence of otorhinolaryngologic infectious diseases decreased during the COVID-19 pandemic period in the Korean population. In addition, the seasonality of otorhinolaryngologic infectious disease was diminished during the COVID-19 pandemic. NPIs may control the infection rate and epidemics of infectious diseases during the COVID-19 era.

## Figures and Tables

**Table 1:** Mean and standard deviation of the incidence of diseases before and during COVID-19 and their difference.

Diseases	Before COVID-19		During COVID-19		*p* Values of Difference	
	Mean	SD	Mean	SD	Mean	Variance
URI	1,819,884.7	381,893.5	792,754.8	154,661.7	<0.001 *	0.008 †
Influenza	221,636.5	350,137.6	1776.9	1167.4	<0.001 *	0.048 †
Acute tonsillitis	659,617.9	118,633.2	279,773.2	49,945.3	<0.001 *	0.008 †
Peritonsillar abscess	86,037.4	15,697.1	36,877.9	5960.2	<0.001 *	0.008 †
Retropharyngeal and parapharyngeal abscess	57,056.0	11,596.6	24,739.4	4299.3	<0.001 *	0.008 †
Acute laryngitis and bronchitis	419,958.0	100,744.8	181,879.4	31,304.8	<0.001 *	0.008 †
Stomatitis and related lesions	102,796.3	10,744.6	84,670.7	6224.4	<0.001 *	0.077
Acute sinusitis	539,828.7	124,631.7	243,105.9	58,250.4	<0.001 *	0.030 †
Rhinitis	1,028,775.8	238,879.4	675,294.8	170,040.4	<0.001 *	0.055
Otitis media	343,229.7	55,749.3	148,861.7	23,754.1	<0.001 *	0.008 †
Dizziness	104,910.8	10,232.1	96,040.7	10,232.2	0.022 *	0.537

* Mann—Whitney U test, significance at <0.05; † Levene’s test in nonparametric data, significance at <0.05.

**Table 2:** Mean and standard deviation of the incidence of diseases before and during COVID-19 and their differences in the subgroups relative to sex.

Diseases	Before COVID-19		During COVID-19		*p* Values of Difference	
	Mean	SD	Mean	SD	Mean	Variance
**Men**						
URI	808,009.1	166,143.8	345,512.2	67,529.2	<0.001 *	0.008 †
Influenza	102,389.1	161,148.9	817.0	540.9	<0.001 *	0.074
Acute tonsillitis	292,892.4	50,747.4	119,724.5	20,979.9	<0.001 *	0.008 †
Peritonsillar abscess	38,552.1	6811.1	16,007.3	2472.9	<0.001 *	0.008 †
Retropharyngeal and parapharyngeal abscess	24,954.4	4882.1	10,892.8	1827.7	<0.001 *	0.008 †
Acute laryngitis and Bronchitis	179,642.1	43,109.3	78,923.5	13,443.6	<0.001 *	0.008 †
Stomatitis and related lesions	46,071.0	4914.4	37,484.5	2251.5	<0.001 *	0.069
Acute sinusitis	257,029.9	57,383.9	121,754.1	29,431.8	<0.001 *	0.038 †
Rhinitis	493,887.1	113,755.6	340,218.0	87,028.1	<0.001 *	0.134
Otitis media	169,809.7	27,017.7	72,879.5	11,794.7	<0.001 *	0.008 †
Dizziness	37,218.4	4031.7	34,242.0	3812.3	0.043 *	0.610
**Women**						
URI	1,011,875.6	216,322.6	447,242.6	87,408.5	<0.001 *	0.006 †
Influenza	119,247.3	189,106.3	959.9	627.6	<0.001 *	0.037 †
Acute tonsillitis	366,725.5	68337.2	160,048.6	29,027.1	<0.001 *	0.006 †
Peritonsillar abscess	47,485.3	8947.4	20,870.6	3507.1	<0.001 *	0.006 †
Retropharyngeal and parapharyngeal abscess	32,101.6	6739.1	13,846.5	2491.6	<0.001 *	0.006 †
Acute laryngitis and bronchitis	240,315.9	57,726.4	102,955.8	17,975.3	<0.001 *	0.006 †
Stomatitis and related lesions	56,725.3	5947.9	47186.2	4027.3	<0.001 *	0.035 †
Acute sinusitis	282,798.8	67,455.3	121,351.8	28,910.2	<0.001 *	0.020 †
Rhinitis	534,888.8	125,704.1	335,076.8	83,425.7	<0.001 *	0.333
Otitis media	173,419.9	28,775.7	75,982.2	11,992.1	<0.001 *	0.006 †
Dizziness	67,692.4	6259.8	61,798.7	6524.2	0.008 *	0.926

* Mann—Whitney U test, significance at <0.05; † Levene’s test in nonparametric data, significance at <0.05.

**Table 3:** Mean and standard deviation of the incidence of diseases before and during COVID-19 and their differences in the subgroups relative to age.

Diseases	Before COVID-19		During COVID-19		*p* Values of Difference	
	Mean	SD	Mean	SD	Mean	Variance
**Age 0–19 years old**						
URI	718,215.0	128,970.4	316,330.5	115,637.1	<0.001 *	0.022 †
Influenza	118,451.5	180,263.3	609.6	412.3	<0.001 *	0.050
Acute tonsillitis	248,149.3	48,522.3	97,814.2	32,298.2	<0.001 *	0.026 †
Peritonsillar abscess	25,512.7	4769.3	10281.5	3223.4	<0.001 *	0.023 †
Retropharyngeal and parapharyngeal abscess	19,341.0	3642.8	7731.1	2781.8	<0.001 *	0.012 †
Acute laryngitis and bronchitis	110,269.8	27,491.9	41,969.3	14,762.1	<0.001 *	0.017 †
Stomatitis and related lesions	23,150.9	8331.2	16,477.1	1903.5	<0.001 *	0.475
Acute sinusitis	290,623.6	67,944.1	131,921.8	50,112.8	<0.001 *	0.143
Rhinitis	466,588.7	109,804.7	302,083.9	103,858.1	<0.001 *	0.569
Otitis media	227,843.8	49,043.2	68,770.6	20,508.9	<0.001 *	0.008 †
Dizziness	5336.8	1149.6	4584.1	1436.5	0.089	0.210
**Age 20–59 years old**						
URI	784,291.7	227,502.8	311,720.8	53,937.4	<0.001 *	0.008 †
Influenza	85,726.2	147,894.7	814.1	582.5	<0.001 *	0.124
Acute tonsillitis	328,817.8	77,712.5	138,490.6	20,547.8	<0.001 *	0.008 †
Peritonsillar abscess	46,454.8	10,447.3	19,399.9	2918.4	<0.001 *	0.008 †
Retropharyngeal and parapharyngeal abscess	29,282.9	7288.1	12,392.9	2123.3	<0.001 *	0.012 †
Acute laryngitis and bronchitis	221,135.6	59,408.4	91,148.6	16,522.8	<0.001 *	0.008 †
Stomatitis and related lesions	44,485.5	3424.8	35,926.8	2579.5	<0.001 *	0.052
Acute sinusitis	198,909.4	52,964.1	84,172.0	14,543.4	<0.001 *	0.012 †
Rhinitis	412,045.3	111,175.6	259,300.3	73,503.3	<0.001 *	0.151
Otitis media	72,384.2	7520.6	44,657.5	3794.8	<0.001 *	0.008 †
Dizziness	44,766.6	4387.5	39478.7	4007.3	0.001 *	0.993
**Age 60+ years old**						
URI	317,955.5	106,564.2	164,960.2	29,720.8	<0.001 *	0.019 †
Influenza	17,514.0	34,873.4	353.5	191.7	<0.001 *	0.204
Acute tonsillitis	82,870.0	22,396.6	43,566.0	5408.0	<0.001 *	0.030 †
Peritonsillar abscess	14,101.9	3850.2	7210.1	1037.9	<0.001 *	0.023 †
Retropharyngeal and parapharyngeal abscess	8451.3	2324.4	4626.0	659.8	<0.001 *	0.045 †
Acute laryngitis and bronchitis	88,750.5	23,658.4	48,867.8	6315.1	<0.001 *	0.027 †
Stomatitis and related lesions	35,194.9	2313.6	32,297.7	2474.2	0.002 *	0.581
Acute sinusitis	50,521.8	14,750.5	27,119.8	3757.3	<0.001 *	0.029 †
Rhinitis	150,577.6	40,633.5	114,227.2	20,862.3	0.004 *	0.013 †
Otitis media	43,161.5	2499.3	35,538.9	2120.9	<0.001 *	0.012 †
Dizziness	54,853.7	5045.5	52,020.8	4923.0	0.101	0.679

* Mann—Whitney U test, significance at <0.05; † Levene’s test in nonparametric data, significance at <0.05.

## Data Availability

Releasing of the data by the researcher is not legally permitted. All the data are available from the database of the Korea Centers for Disease Control and Prevention. The Korea Centers for Disease Control and Prevention allows data access, at a particular cost, for any researcher who promises to follow research ethics. The data of this article can be downloaded from the website after agreeing to follow the research ethics.
